# Gosling Designer: a Platform to Democratize Construction and Sharing of Genomics Data Visualization Tools

**Published:** 2025-10-28

**Authors:** Sehi L’Yi, John Conroy, Priya Misner, David Kouřil, Lisa Choy, Nezar Abdennur, Nils Gehlenborg

**Affiliations:** 1 Harvard Medical School, Boston, MA, USA; 2 UMass Chan Medical School, Worcester, MA, USA

## Abstract

Analysis of genomics data is central to nearly all areas of modern biology. Despite significant progress in artificial intelligence (AI) and computational methods, these technologies require significant human oversight to generate novel and reliable biological insights. Consequently, the genomics community has developed a substantial number of diverse visualization approaches^1^ and a proliferation of tools that biologists rely on in their data analysis workflows^2^. While there are a few commonly used visualization tools for genomics data, such as IGV^3^, Circos^4^, or JBrowse^5^, many tools target specific use cases for genomics data interpretation and offer only a limited, predefined set of visualization types. Moreover, static visualizations—which are the output of the majority of existing tools—often fail to support exploratory analysis. Developing interactive visualizations and tools typically requires significant time and technical expertise, even when supported by modern LLM-powered coding assistants, and the resulting visualizations can be difficult to share among collaborators.

We developed Gosling Designer, an all-in-one platform for editing, exploring, and sharing visualizations of genomics data (Figure 1). Gosling Designer addresses four key challenges observed in existing genomics visualization tools: (1) limited versatility, (2) difficulty of visualization authoring, (3) complexity of data management, and (4) barriers to sharing and collaboration.

Gosling Designer builds on the Gosling grammar^6^, enabling the creation of a broad spectrum of genome-mapped data visualizations tailored to diverse analysis use cases. It supports not only the creation of conventional genomics visualizations but also the design of novel depictions driven by emerging genomics technologies. In addition to visualizing genome sequences in two-dimensional (2D) space using linear and circular layouts, Gosling Designer also allows three-dimensional (3D) representations, such as the physical structure of genomes. Through this expressive power of Gosling Designer, users can create classic genome browser tracks, ideograms, genome interaction matrices, interactive Circos-like plots^4^, and 3D genome structures. Visualizations created in Gosling Designer incorporate powerful built-in user interactions, including seamless zooming and panning, and support linked multiple views for effective analysis. This further enables combining views for effective data exploration, such as a Chromoscope^7^ visualization that combines a Circos plot as a whole genome overview with linked genome browser tracks for showing narrow genomic regions across scales.

Gosling Designer democratizes genomics data visualization by providing a graphical interface that enables a broad range of genomics stakeholders—including experimentalists, clinicians, computational biologists, and software developers—to create customized visualizations with ease. Through familiar user interactions, such as drag and drop, users can construct complex multi-view visualizations without programming. It offers an extensive set of starter visualization examples and track templates that allow users to quickly adapt designs to their own data or switch between alternative visualization options within a gallery. Gosling Designer’s flexible visual mapping, a paradigm familiar from general-purpose visualization tools, allows fine-grained control over how visual representations are designed (e.g., selecting mark types or adjusting visual channels). For advanced users, an integrated code editor exposes the underlying Gosling grammar, providing full programmatic control. The aforementioned user interfaces—templates, visual mapping, and code editor—are linked bidirectionally, lowering users’ learning curve and facilitating iterative design, as demonstrated in prior visualization research^8^. Finally, Gosling Designer records interaction histories and presents them in an interactive view, enabling users to easily explore, undo, or revisit previous visualization states.

Gosling Designer includes an integrated data management platform that streamlines the organization and reuse of genomics data sources for visualization. Users can create accounts to organize genomics files in their workspaces. Users can create personal or shared workspaces to add and manage references to publicly accessible genomics data source, e.g., in community data repositories and associated metadata, such as genome assemblies and assay types. This data structure facilitates efficient data discovery through metadata- or keyword-based filtering, allowing users to locate datasets of interest for visualization. Gosling Designer supports a broad range of standard genomics file formats (e.g., BigWig, BED, GFF, BAM, VCF, CSV/TSV), enabling seamless visualization without the need for additional data preprocessing.

Gosling Designer supports collaborative construction of genomics data visualizations—a critical need in genomics analysis that is often unmet by existing tools^2^. Users can invite collaborators to shared workspaces with configurable access levels (viewer, editor, or administrator), allowing joint management and editing of visualizations and data sources. For streamlined sharing, users can generate private links that are viewable by shared users only, providing direct access to previously created visualizations. All visualizations are fully compatible with the broader Gosling ecosystem, enabling exported specifications to be seamlessly reused in Python notebooks through Gos^9^ or integrated into interactive web applications via Gosling.js^6,9^.

By combining flexibility, usability, and collaboration, Gosling Designer democratizes the creation of both established and novel genome-mapped data visualizations. This broadens participation in visualization design and empowers diverse users to more effectively interpret, analyze, and communicate complex genomics data. The use of the Gosling grammar provides flexibility in extending Gosling Designer for additional features. We envision that the Gosling Designer platform will play an important role in efficient human-AI collaboration for analysis of genomic data.

## Figures and Tables

**Figure 1.**
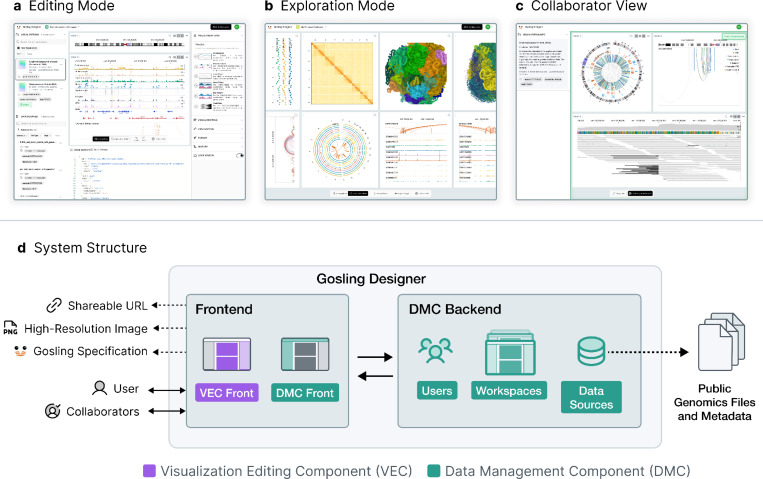
Overview of the user interfaces and system structure of Gosling Designer. **a**, The interface in *editing* mode with genome browser tracks. **b**, The interface in *exploring* mode, displaying a genome interaction matrix with a 3D genome view. **c**, A Chromoscope^7^ visualization created using Gosling Designer that is shared with a collaborator. **d**, The schematic diagram showing the backend-frontend model of Gosling Designer with the visualization editing component and the data management component.
